# Draft Genome Sequences of Three European Laboratory Derivatives from Enterohemorrhagic *Escherichia coli* O157:H7 Strain EDL933, Including Two Plasmids

**DOI:** 10.1128/genomeA.01331-15

**Published:** 2016-04-07

**Authors:** Lea Fellner, Christopher Huptas, Svenja Simon, Anna Mühlig, Siegfried Scherer, Klaus Neuhaus

**Affiliations:** a Lehrstuhl für Mikrobielle Ökologie, Wissenschaftszentrum Weihenstephan, Technische Universität München, Freising, Germany; b Lehrstuhl für Datenanalyse und Visualisierung, Fachbereich Informatik und Informationswissenschaft, Universität Konstanz, Konstanz, Germany

## Abstract

*Escherichia coli* O157:H7 EDL933, isolated in 1982 in the United States, was the first enterohemorrhagic *E. coli* (EHEC) strain sequenced. Unfortunately, European labs can no longer receive the original strain. We checked three European EDL933 derivatives and found major genetic deviations (deletions, inversions) in two strains. All EDL933 strains contain the cryptic EHEC-plasmid, not reported before.

## GENOME ANNOUNCEMENT

Enterohemorrhagic *Escherichia coli* (EHEC) O157:H7 strain EDL933 (ATCC 43895), isolated in 1982, was the first EHEC strain to be sequenced in 2001 (1). In the same year, the genome of EHEC strain Sakai was published (2). The large 92-kb-plasmid pO157 was published in 1998 (3). Because the first EDL933 sequence was inferior, it was recently resequenced (4). Despite its frequent use in research, neither the strain nor its DNA are available to European researchers due to export restrictions of the United States. Unfortunately, EHEC tends to undergo genomic rearrangements (5), which often remain unnoticed.

We sequenced and compared three European derivatives of EDL933. The first derivative, CIP 106327 (Collection de l’Institute Pasteur, Paris, France), was obtained in 2003, freeze-dried after a single sub-cultivation, and deposited in our Weihenstephan strain collection as WS4202. The next derivative WS4435 (= BFEL E135) from R. Pichner (Max-Rubner Institut, Kulmbach, Germany) should be a direct derivative of ATCC 43895. The third derivative, WS4678, was donated by H. Schmidt (Universität Hohenheim, Germany), who received it from L. Beutin (Bundesinstitut für Risikobewertung, Berlin, Germany).

DNA was isolated using CTAB (6) and fragmented using a Covaris E220. Adapter sequences were added using the TruSeq DNA sample preparation kit (Illumina). Libraries were sequenced on a MiSeq station after preparation using the Illumina MiSeq reagent kit v2 (read length 2 × 150) according to the manual. DNA of WS4202 was additionally sequenced on a Pacific Bioscience PacBio RS II (GATC Biotech AG, Konstanz, Germany) for ring closure. Reads from the Illumina-based sequencing were mapped to the original EHEC genome and plasmid sequence (GenBank accession numbers NC_002655 and NC_007414) using Bowtie2 version 2.0.5 (7), considering reads with a mapping quality ≥99% and a base quality ≥99%. In WS4202 and WS4678 we found a mega-inversion of about 1.4 Mbp and larger regions, including OI#48, were missing. After further analysis using the primer system of Bielaszewska et al. (5), OI#48 appeared to be deleted in WS4202 and WS4678. Only the genome of WS4435 appeared to be comparable to the original strain, i.e., similar to the sequence published by Latif et al. (4).

Genomic rearrangements of EHEC have been observed before (5, 8, 9). In the similar strain Sakai, deletions in various prophages were found (10, 11). Chances are good that derivates possess rearranged genomes and, hence, changed phenotypes (5). This should be kept in mind before choosing strains to conduct experiments.

In a recent genome update (4), the cryptic EHEC-plasmid has gone unnoticed again for EDL933. We assembled unmapped reads using ABySS v1.3.3 (12). A *de novo* contig exactly matched pOSAK1 (3.3 kbp, NC_002127) (2). However, the original pO157 of EDL933 was found to be incomplete, 636 bp are missing. The combined ratio (i.e., copy number) of chromosome:pO157:pOSAK1 for all three European strains is approximately 1:2:30.

### Nucleotide sequence accession numbers.

The genome sequences were deposited in GenBank (see Table 1). The versions for WS4202 and WS4678 described in this paper are the first versions; for WS4435 it is the second version.

**TABLE 1:** Genome accession numbers

Strain	Accession no.	Local ID	No. of contigs	Sequence length (bp)
WS4202	CP012802	genome-WS4202	1	5,332,063
WS4202	CP012803	pO157-WS4202	1	92,739
WS4202	CP012804	pOSAK1-WS4202	1	3,306
WS4435	LKAK00000000	Whole genome shotgun	143	
WS4678	LKAL00000000	Whole genome shotgun	195	
